# Asymptomatic Bacteriuria in Post Renal Transplant Patients: To Treat or Not?

**DOI:** 10.7759/cureus.15713

**Published:** 2021-06-17

**Authors:** Anas O Almaimani

**Affiliations:** 1 Department of Urology, King Abdulaziz Airbase Hospital, Dhahran, SAU

**Keywords:** bacteriuria, kidney transplantation, antibiotics, urinary tract infection, renal transplant surgery

## Abstract

Urinary tract infections (UTIs) can commonly occur within the first few weeks following kidney transplantation procedures. Although the management of asymptomatic bacteriuria and acute graft pyelonephritis is important to reduce the risk of graft rejections following kidney transplantation, the efficacy of antibiotics administration remains controversial among studies in the literature. The aim of this review is to elaborate more on asymptomatic bacteriuria following kidney transplantation and try to formulate proper evidence about the efficacy of antibiotics administration on eliminating the frequency of infections and enhancing the quality of care for patients. Most studies in the literature are observational, which are usually biased in the interventions. However, the current evidence regarding the management and screening of asymptomatic bacteriuria seems to discourage such an approach. Almost all of the included studies reported that antibiotic administration did not significantly lower the rates of secondary symptomatic UTIs or enhance the functions of the graft. In addition, there is no significant impact on mortality and other clinical outcomes. Lastly, the frequent administration of antibiotics can significantly increase the risk of recurrence due to the emergence of novel strains of bacteria that are resistant to the currently administered antibiotics making it unfavorable.

## Introduction and background

Urinary tract infections (UTIs) can commonly occur within the first few weeks following kidney transplantation procedures, with an estimated incidence of 6%-83% as reported in the literature [1-5]. UTIs can occur as symptomatic or asymptomatic infections and can lead to acute pyelonephritis, which is a serious complication [5]. Secondary to acute pyelonephritis, studies have demonstrated that secondary graft dysfunction or even rejection might occur leading to hospitalization [6,7]. Previous studies have demonstrated that acute graft pyelonephritis might be associated with chronic graft dysfunctions or rejections, increased incidence of cytomegalovirus infection, and increased risk of mortality [4,5,8-11].

Recently, the rate of UTIs is increasing and the risk of catching an infection by multi-drug resistant organisms has drawn much attention due to the potentially serious complications that may occur in patients post renal transplantation procedure with a high-risk incidence of recurrence [12-14]. Moreover, more concerns are reported as the management of multi-drug resistant organisms require the administration of the new generations of antibiotics. As a result, more side effects can arise including nephrotoxicity, which can worsen the prognosis of the graft and might require the administration of calcineurin inhibitors [15,16]. Therefore, it has been suggested that efforts should be directed to reduce the levels of drug resistance to enhance the management and increase the quality of care.

Although the management of asymptomatic bacteriuria and acute graft pyelonephritis is important to reduce the risk of graft rejections following kidney transplantation, the efficacy of antibiotics administration remains controversial among studies in the literature [17,18]. As a result of the absent management guidelines, treatment of asymptomatic bacteriuria is not consistent among transplant centers and healthcare facilities [19-24]. However, recent guidelines suggest the importance of early screening and management of bacteriuria within the first weeks after kidney transplantation [23,25]. The screening and management of asymptomatic bacteriuria should be early as a previous randomized controlled trial reported that antibiotics administration was not successful in reducing the frequency of UTIs as the treatment modalities were prescribed within the second month from transplantation [26]. The aim of the present study is to elaborate more on asymptomatic bacteriuria following kidney transplantation and provide proper evidence about the efficacy of antibiotics administration on eliminating the frequency of infections and enhancing the quality of care for patients with recent kidney transplantation.

## Review

Methodology

An extensive literature search of the PUBMED, Web of Science, Medline, Cochrane, and EMBASE databases was performed using the medical combination of all possible related terms. Papers discussing risk factors of asymptomatic bacteriuria and information about different management approaches were screened for relative information. A final drafting of the manuscript was performed based on the personal opinion of the author to draft this paper.

Risk factors for asymptomatic bacteriuria

Many risk factors that have been reported among studies in the literature are mainly for bacteriuria and were not specified to asymptomatic bacteriuria solely. A previous investigation reported that females who have a delay in removal of a Foley catheter with prolonged function of the graft are associated with bacteriuria following kidney transplantation [27]. It has been estimated that catheters increase the risk of developing bacteriuria by 5%. Moreover, more than two-third of patients usually develop bacteriuria following two weeks from catheterization [28]. Among the studies that reported the risk factors to catch asymptomatic bacteriuria as a sole risk factor, a previous study reported that having a positive Charlson comorbidity index and being female were the only predictors to developing the infection [29]. The primary presence of glomerulonephritis in renal transplantation is also a significant predictor for developing asymptomatic bacteriuria [30].

To treat or not?

Whether to treat asymptomatic bacteriuria in patients that underwent renal transplantation is still controversial among clinicians and transplantation centers. A previous survey by Coussement et al. [19] was conducted to assess the current practice and attitudes towards the screening and management of asymptomatic bacteriuria in patients with kidney transplantations in Europe. The authors reported that the screening and management practices were commonly practiced according to the guidelines, irrespective of being aware of the efficacy or benefits and adverse events that could potentially result from such practices.

Coussement et al. [17] reported that the evidence regarding the efficacy of antibiotics administration in patients suffering from asymptomatic bacteriuria following renal transplantation is not sufficient to draw any solid conclusions and that additional prospective studies with adequate interventions and randomization are needed. However, the authors relied on such results from four studies, including one prospective case-control study [31], and another three retrospective observational studies [30,32,33]. In the case-control study, no significant differences were found between the two study groups in terms of risk of developing symptomatic infections or other adverse events [31]. In the same context, the other three retrospective observational studies also concluded that antibiotic management did not significantly lower the incidence of symptomatic UTIs. The rate of bacterial resistance was observed in many patients with asymptomatic bacteriuria that were treated with antibiotics, and that the infections are usually self-limited [30,32,33]. The same findings were also reported in a previous 2015 review by Singh et al. [34] that also relied on data from these studies and also called for future observational studies to be conducted to formulate solid evidence regarding whether to administer antibiotics for treating patients with symptomatic bacteriuria following kidney transplantation to enhance the outcomes and reduce the risk of developing adverse events.

In a randomized multicenter observational trial, Sabé et al. [35] recruited 205 patients that underwent kidney transplantation to assess the efficacy of antibiotic therapy on the prognosis of the infections among these patients in the first year following renal transplantation. They reported that no significant differences were found between the two study groups in terms of acute pyelonephritis, and other secondary endpoints as graft rejection of dysfunction, hospitalization, and mortality. Accordingly, they suggested that systemic antibiotic therapy should not be recommended for these patients due to the absence of efficacy and the potential development of bacterial resistance to the administered antibiotics. In another randomized controlled trial, Origuen et al. [26] reported that screening and management of asymptomatic bacteriuria in patients that underwent renal transplantation did not significantly affect the prognosis and development of adverse events in these patients as compared to the control group.

Another observational study by Bohn et al. [36] was conducted to assess the incidence of asymptomatic bacteriuria among patients that underwent kidney transplantation and whether the administration of antibiotics was efficacious in eliminating the infection and enhancing the prognosis or not. They reported that among 527 patients, the incidence was 12.1%, of which, three-fourths were treated were systemic antibiotics. However, the authors reported that the occurrence of asymptomatic bacteriuria within the first month following transplantation did not increase the risk of potential adverse events. Moreover, antibiotics management was not efficacious in controlling and eradicating the infections and should not be used in these patients to prevent the creation of multi-drug resistant organisms.

A previous retrospective pediatric study by Bonnéric et al. [37] reported that systematic antibiotic therapy for patients undergoing kidney transplantation is not recommended for the prevention of asymptomatic bacteriuria. The authors reported that acute pyelonephritis or lower UTIs occurred less frequently with untreated asymptomatic bacteriuria. These results were supported by Arencibia et al. [38] who showed that most patients with asymptomatic bacteriuria usually have spontaneous bacterial clearance and that antibiotics administration can lead to the emergence of new resistant bacterial strains.

On the other hand, another retrospective analysis of 276 kidney transplantation patients by Kotagiri et al. [39] showed that asymptomatic bacteriuria was present in 27% of them, and treatment of asymptomatic bacteriuria can reduce the risk of developing symptomatic UTIs. However, the authors reported that the presence of bacteriuria or UTIs was not significantly associated with the development of adverse events and complications as estimated along one year following renal transplantation. Moreover, the previous observational study by Gołębiewska et al. [29] reported that further evidence about the administration of antibiotics for treating asymptomatic bacteriuria is still needed as they noticed that only a small percentage of symptomatic UTIs occurred by the same organisms that caused the asymptomatic bacteriuria in their population, and that recurrence could be associated with repeated and frequent use of antibiotics.

A previous meta-analysis by Gómez-Ochoa et al. [40] was conducted to estimate the incidence of asymptomatic bacteriuria and the efficacy of treatment modalities on enhancing the outcomes. They reported that according to the 15 included studies, the incidence of asymptomatic bacteriuria increased from 22% to 30% from the first month to the first year following kidney transplantation. Moreover, they reported that management of asymptomatic bacteriuria was not significantly associated with reduced risk of developing symptomatic UTIs, or graft dysfunction as estimated by the renal functions, which were not significantly impacted, according to eight included studies in the analysis. Another systematic review was published by Coussement et al. [18] analyzed the results from randomized controlled trials only and reported that antibiotic therapy for managing asymptomatic bacteriuria showed uncertain results about preventing the development of symptomatic UTIs, all-cause mortality, graft rejections, adverse rejections, or serum creatinine levels. Therefore, the authors concluded that the evidence for whether recommending for or against the administration of antibiotics in patients with renal transplantation was poor, and the authors called for future investigations.

In contrast to the recommendation of the study by Coussement et al. [18], the Infectious Diseases Society of America recommended against the screen and treat strategy for asymptomatic bacteriuria in patients that had kidney transplantation. They support their recommendations by the poor evidence from the randomized trials and the previously published observational studies [25]. However, Coussement and colleagues replied to this report by saying that the evidence that these guidelines were built on is not sufficient as it was based on observational studies, which usually lack proper interventions, and only two randomized trials, which were reported to be of low quality with insufficient sample size to formulate sufficient evidence [41].

In 2021, a randomized, multicenter controlled trial was published by Coussement et al. [42] also to assess the efficacy of treating asymptomatic bacteriuria with systemic antibiotics to reduce the rates of development of symptomatic complications. They reported that antibiotics administration in 100 patients with kidney transplantation did not significantly reduce the rate of complications and symptomatic adverse events among these patients as compared to the other 99 patients that did not receive antibiotic therapy, as reported in their population that developed asymptomatic bacteriuria in at least two months following kidney transplantation. Moreover, the authors found that among the group that was recruited to receive the antibiotic therapy, the frequency of antibiotics administration significantly increased, and therefore, the risk of emergence of multi-drug resistance bacteria also increased.

## Conclusions

Most studies in the literature are observational, which are usually biased in the interventions. However, the current evidence regarding the management and screening of asymptomatic bacteriuria after renal transplantation seems to discourage anti-bacterial management. Antibiotic administration did not significantly lower the rates of secondary symptomatic UTIs or enhance the functions of the graft. In addition, it did not have a significant impact on mortality and other clinical outcomes. Future prospective randomized studies and clinical trials on a larger scale are encouraged to support the current evidence and help formulate sufficient guidelines for clinicians and transplantation centers.

## References

[1] Sorto R, Irizar SS, Delgadillo G, Alberú J, Correa-Rotter R, Morales-Buenrostro LE (2010). Risk factors for urinary tract infections during the first year after kidney transplantation. Transplant Proc.

[2] Säemann M, Hörl WH (2008). Urinary tract infection in renal transplant recipients. Eur J Clin Invest.

[3] Muñoz P (2001). Management of urinary tract infections and lymphocele in renal transplant recipients. Clin Infect Dis.

[4] Valera B, Gentil MA, Cabello V, Fijo J, Cordero E, Cisneros JM (2006). Epidemiology of urinary infections in renal transplant recipients. Transplant Proc.

[5] Fiorante S, Fernández-Ruiz M, López-Medrano F (2011). Acute graft pyelonephritis in renal transplant recipients: incidence, risk factors and long-term outcome. Nephrol Dial Transplant.

[6] Linares L, García-Goez JF, Cervera C (2009). Early bacteremia after solid organ transplantation. Transplant Proc.

[7] Silva M Jr, Marra AR, Pereira CA, Medina-Pestana JO, Camargo LF (2010). Bloodstream infection after kidney transplantation: epidemiology, microbiology, associated risk factors, and outcome. Transplantation.

[8] Dupont PJ, Manuel O, Pascual M (2010). Infection and chronic allograft dysfunction. Kidney Int Suppl.

[9] Pellé G, Vimont S, Levy PP (2007). Acute pyelonephritis represents a risk factor impairing long-term kidney graft function. Am J Transplant.

[10] Kamath NS, John GT, Neelakantan N, Kirubakaran MG, Jacob CK (2006). Acute graft pyelonephritis following renal transplantation. Transpl Infect Dis.

[11] Lorenz EC, Cosio FG (2010). The impact of urinary tract infections in renal transplant recipients. Kidney Int.

[12] Origüen J, Fernández-Ruiz M, López-Medrano F (2016). Progressive increase of resistance in Enterobacteriaceae urinary isolates from kidney transplant recipients over the past decade: narrowing of the therapeutic options. Transpl Infect Dis.

[13] Bodro M, Sanclemente G, Lipperheide I (2015). Impact of antibiotic resistance on the development of recurrent and relapsing symptomatic urinary tract infection in kidney recipients. Am J Transplant.

[14] Alevizakos M, Nasioudis D, Mylonakis E (2017). Urinary tract infections caused by ESBL-producing Enterobacteriaceae in renal transplant recipients: a systematic review and meta-analysis. Transpl Infect Dis.

[15] Cervera C, van Delden C, Gavaldà J, Welte T, Akova M, Carratalà J (2014). Multidrug-resistant bacteria in solid organ transplant recipients. Clin Microbiol Infect.

[16] Winthrop KL, Mariette X, Silva JT (2018). ESCMID Study Group for Infections in Compromised Hosts (ESGICH) Consensus Document on the safety of targeted and biological therapies: an infectious diseases perspective (soluble immune effector molecules [II]: agents targeting interleukins, immunoglobulins and complement factors). Clin Microbiol Infect.

[17] Coussement J, Abramowicz D (2014). Should we treat asymptomatic bacteriuria after renal transplantation?. Nephrol Dial Transplant.

[18] Coussement J, Scemla A, Abramowicz D, Nagler EV, Webster AC (2018). Antibiotics for asymptomatic bacteriuria in kidney transplant recipients. Cochrane Database Syst Rev.

[19] Coussement J, Maggiore U, Manuel O (2018). Diagnosis and management of asymptomatic bacteriuria in kidney transplant recipients: a survey of current practice in Europe. Nephrol Dial Transplant.

[20] Kidney Disease: Improving Global Outcomes (KDIGO) Transplant Work Group (2009). KDIGO clinical practice guideline for the care of kidney transplant recipients. Am J Transplant.

[21] Nicolle LE, Bradley S, Colgan R, Rice JC, Schaeffer A, Hooton TM (2005). Infectious Diseases Society of America guidelines for the diagnosis and treatment of asymptomatic bacteriuria in adults. Clin Infect Dis.

[22] Vidal E, Cervera C, Cordero E (2015). Management of urinary tract infection in solid organ transplant recipients: consensus statement of the Group for the Study of Infection in Transplant Recipients (GESITRA) of the Spanish Society of Infectious Diseases and Clinical Microbiology (SEIMC) and the Spanish Network for Research in Infectious Diseases (REIPI). Enferm Infecc Microbiol Clin.

[23] Goldman JD, Julian K (2019). Urinary tract infections in solid organ transplant recipients: guidelines from the American Society of Transplantation Infectious Diseases Community of Practice. Clin Transplant.

[24] Parasuraman R, Julian K (2013). Urinary tract infections in solid organ transplantation. Am J Transplant.

[25] Nicolle LE, Gupta K, Bradley SF (2019). Clinical practice guideline for the management of asymptomatic bacteriuria: 2019 update by the Infectious Diseases Society of America. Clin Infect Dis.

[26] Origüen J, López-Medrano F, Fernández-Ruiz M (2016). Should asymptomatic bacteriuria be systematically treated in kidney transplant recipients? Results from a randomized controlled trial. Am J Transplant.

[27] Chordia P, Schain D, Kayler L (2013). Effects of ureteral stents on risk of bacteriuria in renal allograft recipients. Transpl Infect Dis.

[28] Pickard R, Lam T, Maclennan G (2012). Types of urethral catheter for reducing symptomatic urinary tract infections in hospitalised adults requiring short-term catheterisation: multicentre randomised controlled trial and economic evaluation of antimicrobial- and antiseptic-impregnated urethral catheters (the CATHETER trial). Health Technol Assess.

[29] Gołębiewska JE, Dębska-Ślizień A, Rutkowski B (2014). Treated asymptomatic bacteriuria during first year after renal transplantation. Transpl Infect Dis.

[30] Fiorante S, López-Medrano F, Lizasoain M (2010). Systematic screening and treatment of asymptomatic bacteriuria in renal transplant recipients. Kidney Int.

[31] Moradi M, Abbasi M, Moradi A, Jalali A, Boskabadi A (2005). Effect of antibiotic therapy on asymptomatic bacteriuria in kidney transplant recipients. Urology Journal.

[32] Green H, Rahamimov R, Goldberg E (2013). Consequences of treated versus untreated asymptomatic bacteriuria in the first year following kidney transplantation: retrospective observational study. Eur J Clin Microbiol Infect Dis.

[33] El Amari EB, Hadaya K, Bühler L (2011). Outcome of treated and untreated asymptomatic bacteriuria in renal transplant recipients. Nephrol Dial Transplant.

[34] Singh R, Geerlings SE, Bemelman FJ (2015). Asymptomatic bacteriuria and urinary tract infections among renal allograft recipients. Curr Opin Infect Dis.

[35] Sabé N, Oriol I, Melilli E (2019). Antibiotic treatment versus no treatment for asymptomatic bacteriuria in kidney transplant recipients: a multicenter randomized trial. Open Forum Infect Dis.

[36] Bohn BC, Athans V, Kovacs CS, Stephany BR, Spinner ML (2019). Impact of asymptomatic bacteriuria incidence and management post-kidney transplantation. Clin Transplant.

[37] Bonnéric S, Maisin A, Kwon T, Deschênes G, Niel O (2019). Asymptomatic bacteriuria in pediatric kidney transplant recipients: to treat or not to treat? A retrospective study. Pediatr Nephrol.

[38] Arencibia N, Agüera ML, Rodelo C (2016). Short-term outcome of untreated versus treated asymptomatic bacteriuria in renal transplant patients. Transplant Proc.

[39] Kotagiri P, Chembolli D, Ryan J, Hughes PD, Toussaint ND (2017). Urinary tract infections in the first year post-kidney transplantation: potential benefits of treating asymptomatic bacteriuria. Transplant Proc.

[40] Gómez-Ochoa SA, Vega-Vera A (2020). Systematic review and meta-analysis of asymptomatic bacteriuria after renal transplantation: incidence, risk of complications, and treatment outcomes. Transpl Infect Dis.

[41] Coussement J, Scemla A, Abramowicz D, Nagler EV, Webster AC (2020). Management of asymptomatic bacteriuria after kidney transplantation: what is the quality of the evidence behind the Infectious Diseases Society of America guidelines?. Clin Infect Dis.

[42] Coussement J, Kamar N, Matignon M (2021). Antibiotics versus no therapy in kidney transplant recipients with asymptomatic bacteriuria (BiRT): a pragmatic, multicentre, randomized, controlled trial. Clin Microbiol Infect.

